# Catheter-Based Techniques for Addressing Atrioventricular Valve Regurgitation in Adult Congenital Heart Disease Patients: A Descriptive Cohort

**DOI:** 10.3390/jcm12144798

**Published:** 2023-07-20

**Authors:** Abdelhak El Bouziani, Lars S. Witte, Berto J. Bouma, Monique R. M. Jongbloed, Daniëlle Robbers-Visser, Bart Straver, Marcel A. M. Beijk, Philippine Kiès, David R. Koolbergen, Frank van der Kley, Martin J. Schalij, Robbert J. de Winter, Anastasia D. Egorova

**Affiliations:** 1 Department of Cardiology, CAHAL, Centre for Congenital Heart Disease Amsterdam-Leiden, Amsterdam University Medical Centres, AMC, Meibergdreef 9, 1105 AZ Amsterdam, The Netherlands; 2 Department of Cardiology, CAHAL, Centre for Congenital Heart Disease Amsterdam-Leiden, Leiden University Medical Centre, 2300 RC Leiden, The Netherlands; 3 Department of Anatomy and Embryology, Leiden University Medical Center, 2300 RC Leiden, The Netherlands; 4 Department of Congenital Cardiothoracic Surgery, CAHAL, Centre for Congenital Heart Disease Amsterdam-Leiden, Leiden University Medical Centre, 2300 RC Leiden, The Netherlands

**Keywords:** adult congenital heart disease (ACHD), transcatheter valve repair, atrioventricular (AV) regurgitation, hybrid, transcatheter edge-to-edge repair (TEER), Cardioband, valve-in-valve (ViV)

## Abstract

Introduction: Increasing survival of adult congenital heart disease (ACHD) patients comes at the price of a range of late complications—arrhythmias, heart failure, and valvular dysfunction. Transcatheter valve interventions have become a legitimate alternative to conventional surgical treatment in selected acquired heart disease patients. However, literature on technical aspects, hemodynamic effects, and clinical outcomes of percutaneous atrioventricular (AV) valve interventions in ACHD patients is scarce. Method: This is a descriptive cohort from CAHAL (Center of Congenital Heart Disease Amsterdam-Leiden). ACHD patients with severe AV valve regurgitation who underwent a transcatheter intervention in the period 2020–2022 were included. Demographic, clinical, procedural, and follow-up data were collected from patient records. Results: Five ACHD patients with severe or torrential AV valve regurgitation are described. Two patients underwent a transcatheter edge-to-edge repair (TEER), one patient underwent a valve-in-valve procedure, one patient received a Cardioband system, and one patient received both a Cardioband system and TEER. No periprocedural complications occurred. Post-procedural AV valve regurgitation as well as NYHA functional class improved in all patients. The median post-procedural NYHA functional class improved from 3.0 (IQR [2.5–4.0]) to 2.0 (IQR [1.5–2.5]). One patient died 9 months after the procedure due to advanced heart failure with multiorgan dysfunction. Conclusion: Transcatheter valve repair is feasible and safe in selected complex ACHD patients. A dedicated heart team is essential for determining an individualized treatment strategy as well as pre- and periprocedural imaging to address the underlying mechanism(s) of AV regurgitation and guide the transcatheter intervention. Long-term follow-up is essential to evaluate the clinical outcomes of transcatheter AV valve repair in ACHD patients.

## 1. Introduction

Congenital heart disease (CHD) has an estimated prevalence of about 1% in the general population [1]. CHD patients have demonstrated an increasing survival over the past decades due to improved diagnostic imaging modalities as well as advances in surgical, pharmacological, and transcatheter management strategies. This aging group of CHD patients is, however, frequently confronted with a range of late complications such as arrhythmias, heart failure, and valvular dysfunction [2,3]. Transcatheter valve interventions have developed rapidly during the past decade and have become a legitimate alternative to conventional surgical treatment in selected patients [4]. In contrast to non-CHD adults suffering from functional atrioventricular (AV) valve regurgitation, adults with congenital heart disease (ACHD) are typically younger and often have several mechanisms contributing to AV valve dysfunction. Furthermore, the surgical risk in ACHD patients is increased due to complex anatomy, myocardial fibrosis due to the underlying condition, previous surgical interventions and cannulations, and intrathoracic and pericardial adhesions and collateral vessels. Literature on technical aspects, hemodynamic effects, and clinical outcomes of percutaneous AV valve interventions in CHD patients is scarce and these aspects have only been described in several case reports or series [5,6,7]. This descriptive cohort reports the recent experience of transcatheter valve therapies in complex ACHD patients in a large tertiary referral center.

## 2. Methods

This two-center observational, retrospective cohort study was performed at the departments of Cardiology of the Amsterdam University Medical Center and Leiden University Medical Center, united in the center of Congenital Heart Disease Amsterdam-Leiden (CAHAL). Consecutive ACHD patients with hemodynamically severe AV valve lesions (accessed in accordance with the 2021 ESC/EACTS Guidelines for the management of valvular heart disease) who underwent a catheter-based intervention to address the AV valve dysfunction between 2020 and 2022 were included in this cohort [8]. All patients were discussed by the multidisciplinary heart team which included an ACHD specialist, an interventional and an imaging cardiologist, a congenital cardiothoracic surgeon, a pediatric cardiologist, and a specialized nurse. Demographic, clinical, procedural, and follow-up data were collected from patient medical records.

### Statistical Analysis

Descriptive statistics were utilized to summarize data. Normally distributed continuous data are displayed as mean ± standard deviation (SD) and non-normally distributed continuous data are displayed as median and 95% and the first and third interquartiles [IQ1–IQ3]. Categorical data are presented as numbers. Statistical analyses were performed with IBM SPSS Statistics for Windows, version 28 (IBM Corp., Armonk, NY, USA).

## 3. Results

Five complex ACHD patients (two women), median age 52 years (44–74), with severe or torrential symptomatic AV regurgitation were included in this descriptive cohort; Table 1. Three patients had a morphologically systemic left ventricle and two patients had a systemic right ventricle supporting the systemic circulation. Furthermore, in three out of five patients, the AV valve regurgitation was of the subpulmonary ventricle. All patients had a history of previous cardiac operations with a median of two [1.5–2] operations. At baseline two patients were in NYHA functional class IV, two patients were in NYHA functional class III, and one patient was in NYHA functional class II [2.5–4.0]. Four patients underwent a percutaneous intervention and one patient had a hybrid procedure. Two patients received a transcatheter edge-to-edge repair (TEER), one patient underwent a transcatheter valve-in-valve procedure, one patient received a Cardioband system, and one patient received a Cardioband system as well as a TEER.

**Figure 1:**
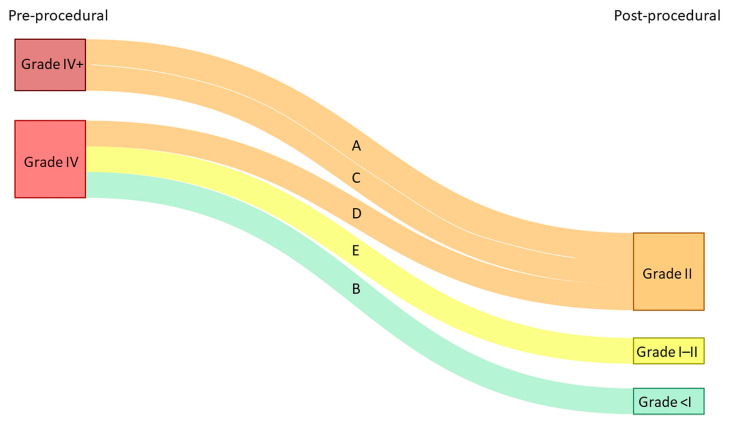
Atrioventricular (AV) valve regurgitation before and after transcatheter valve intervention. Each line represents a single patient (A–E corresponding to patient A–E, respectively).

**Figure 2:**
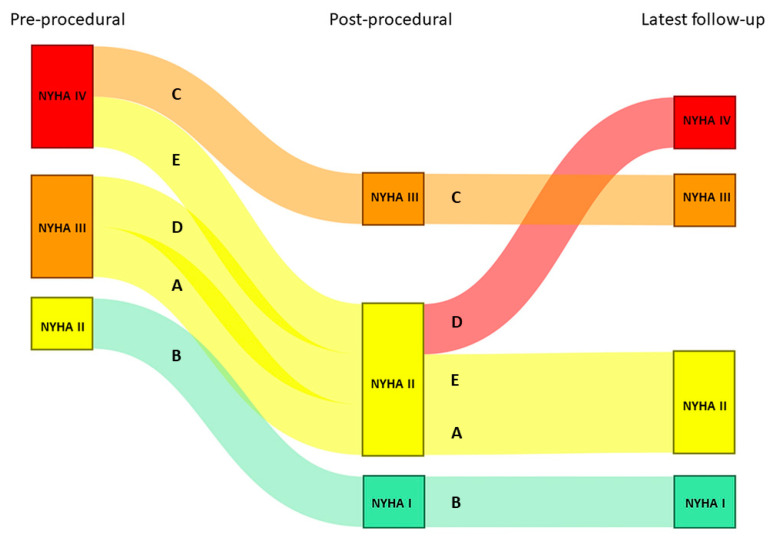
NYHA functional class before and after the transcatheter valve intervention, as well as at latest available follow-up. Each line represents a single patient (A–E corresponding to patient A–E, respectively).

All procedures were performed under general anesthesia with transesophageal (TEE) and/or fluoroscopic guidance, except for one case, which was performed under local anesthesia. No periprocedural complications were reported. Post-procedural AV valve regurgitation improved significantly in all patients (Figure 1). The median follow-up period was 17 months [12.5–23.0]. The median of the post-procedural NYHA functional class was II [1.5–2.5]. At latest follow-up, one patient was in NYHA functional class I, two patients were in NYHA functional class II, and one patient was in NYHA functional class III. One patient (patient D) was in NYHA functional class IV at latest follow-up and died 9 months after the procedure (Figure 2).

### 3.1. Patient A

**Figure 3:**
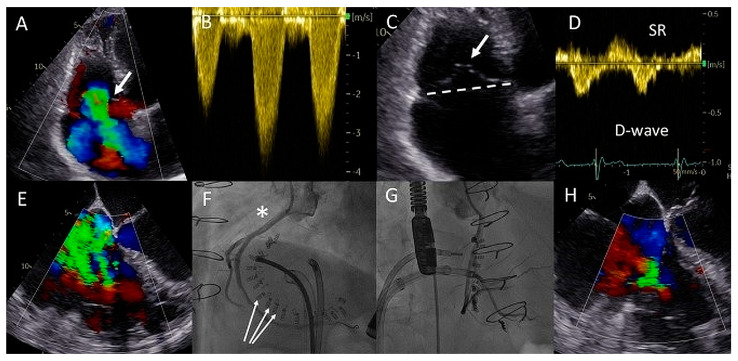
(**A**) Color Doppler apical four-chamber view shows torrential tricuspid regurgitation (TR) with a wide vena contracta (arrow) and flow disturbance filling the enlarged right atrium. (**B**) Continuous wave doppler showing a dense TR signal with elevated right ventricular pressures. (**C**) Noncoaptation of the tricuspid valve (TV) leaflets (arrow) and annulus dilatation (dash line, 53 mm) is seen. (**D**) The hepatic vein Doppler demonstrates a pattern in atrial fibrillation with a prominent and late peaking systolic reversal (SR) wave. The only forward flow is evident in diastole (D-wave). (**E**) Two TR jets (a vena contracta of 4 and 7 mm, respectively, ERO of 90 mm^2^, regurgitant volume of 98 mL) are evident during transesophageal imaging. (**F**) Left anterior oblique and (**G**) right superior oblique fluoroscopic views showing patent right coronary artery (asterisk) and 18 anchors (arrows) between the two TV commissures allowing the Cardioband to significantly reduce the annulus dimensions. (**H**) Transesophageal echocardiography showing significant reduction in TR after the Cardioband annulus reduction procedure (appreciate the difference with panel (**E**), vena contracta of 3 and 5 mm, respectively, ERO 35 mm^2^, regurgitant volume of 41 mL).

A 76-year-old male with a history of a Ross procedure due to severe regurgitation of a bicuspid aortic valve at the age of 25 years, ascending aorta replacement and aortic bioprosthesis implantation at the age of 65 and atrial fibrillation (AF), presented to the emergency department with complaints of dyspnea and progressive weight gain. He was documented to have persistent AF with inadequate rate-control and signs of moderate left- and right-sided decompensation. He was treated with loop diuretics and underwent successful cardioversion to sinus rhythm after recompensation. Transthoracic echocardiography (TTE) revealed moderately reduced left and right ventricular function, normal function of the aortic bioprosthesis (peak gradient < 30 mmHg, mean gradient < 20 mmHg, no regurgitation and pulmonary homograft (peak gradient 25 mmHg, mean gradient 14 mmHg, trivial regurgitation), trivial mitral regurgitation, and torrential tricuspid regurgitation (TR) with elevated filling pressures (peak TR gradient 50 mmHg, estimated right atrial pressures 10–15 mmHg) (Figure 3A–D). Laboratory analysis showed preserved renal function (eGFR 63 mL/min/1.73 m^2^) and elevated levels of NT-proBNP (9652 ng/L, upper reference limit 520 ng/L). Despite pharmacological escalation, the patient remained in NYHA functional class III and experienced frequent recurrences of symptomatic AF. The severe functional TR due to annulus dilatation and progressive decline in systolic function of the overloaded right ventricle was deemed to be the cause. The perioperative risk of surgery involving a third thoracotomy in a septuagenarian was high, and therefore percutaneous options were evaluated. Given the malcoaptation and the dilated tricuspid valve (TV) annulus of 62 × 54 mm (Figure 3C), the heart team deemed transcatheter annular reduction with the Cardioband system (Edwards Lifesciences, Santa Ana, CA, USA) to be the best approach.

The patient underwent a successful Cardioband annular reduction using right venous femoral access under general anesthesia with fluoroscopic (selective cannulation of the right coronary artery) and TEE guidance (Figure 3E–H). A total of 18 anchors were placed between the anteroseptal and posteroseptal commissure reducing the annulus dimensions to 45 × 36 mm. The torrential TR (two jets) was reduced to moderate TR (Figure 3E,H). At two years follow-up, the patient was in NYHA functional class I-II and no heart-failure-related admissions occurred. His NT-proBNP serum levels decreased to 2762 ng/L and at echocardiography he had a stable moderately reduced RV function and moderate TR.

### 3.2. Patient B

**Figure 4:**
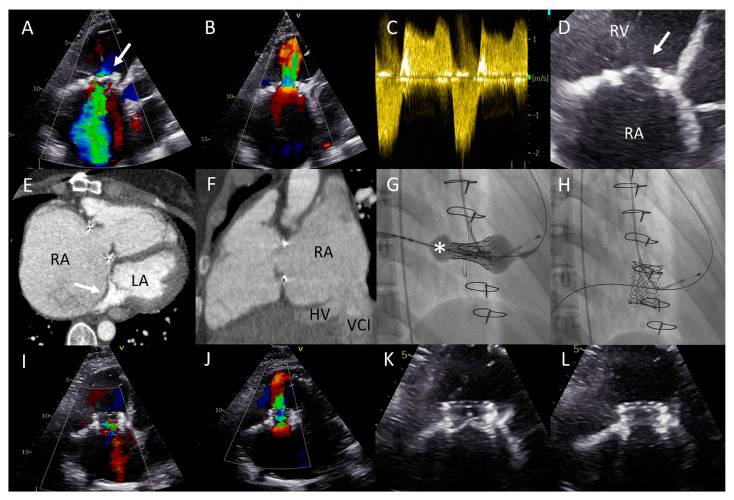
(**A**) Color Doppler apical four-chamber view shows severe tricuspid regurgitation (TR) with a wide vena contracta (arrow) and a systolic jet reaching the roof of the enlarged right atrium (RA). (**B**) Color Doppler showing turbulent inflow through the tricuspid valve (TV) bioprosthesis and aliasing, mean gradient was elevated at 4–5 mmHg. (**C**) Continuous wave Doppler showing a dense TR signal with low velocity. (**D**) Calcified and degenerated tricuspid bioprosthesis (arrow). (**E**) Axial slice through a computed tomography (CT) scan at the level of the right ventricle shows a giant RA and an intra-atrial septum deviation towards the left atrium (arrow), partially suppressing it. (**F**) Sagittal CT slice shows the inflow angle of the inferior vena cava-right RA and the RA-TV. Note the distended hepatic vein (HV). (**G**,**H**) Right anterior oblique fluoroscopy projections show the expansion of the Sapien 3 valve (asterisk) using the ring of the degenerated bioprosthesis as the reference and the final result, respectively. (**I**,**J**) Apical four-chamber color Doppler views showing normal function of the valve-in-valve bioprosthesis (appreciate the difference with (**A**,**B**), respectively). (**K**,**L**) View of the valve-in-valve bioprosthesis in systole (closed) and diastole (open), respectively.

A 40-year-old female born with Ebstein’s anomaly of the TV who had previously undergone a surgical annuloplasty and a TV replacement with a bioprosthesis (TVR, 33 mm Sorin-Pericarbon MoreTM, Sorin Biomedica, Italy) and had two uncomplicated pregnancies afterwards was seen at the outpatient clinic with progressive complaints of reduced exercise tolerance, currently in NYHA functional class II. TTE revealed severe regurgitation and significant stenosis of the TVR bioprosthesis with progressively worsening right ventricular (RV) function and a giant right atrium (RA) partially compressing the left atrial compartment (Figure 4A–E). She was not using any pharmacotherapy, and had preserved renal function and elevated levels of NT-proBNP (670 ng/L, upper reference limit 247 ng/L). Magnetic resonance imaging confirmed preserved left ventricular function (EF 52%) and at least a moderately reduced RV function (EF 40%), likely overestimated in the setting of severe TR (44% regurgitant fraction). Given the significantly reduced RV function and the risks of a third thoracotomy, the heart team deemed a valve-in-valve transcatheter TV implantation to be feasible and lower risk (short-term), allowing the RV function to recover whilst the patient would still be eligible for a surgical re-TVR after the failure of this valve-in-valve prothesis in the future. As a mother of two young children, the patient opted to defer surgery and pursue a transcatheter valve procedure.

This was successfully performed under local anesthesia, by means of right femoral access and fluoroscopy and TTE guidance. A temporary pacemaker wire was placed in the left ventricle and the AgilisTM steerable introducer (Abbott, IL, USA) was used to safely access the RA, given the relatively sharp angle between the vena cava-RA and the RA-RV axis (Figure 4F). The Edwards eSheath TM (Edwards Lifesciences, USA) was then used to implant a 29 mm Edwards SAPIEN 3 valve (Edwards Lifesciences) in the TVR bioprosthesis under rapid pacing (Figure 4G,H). Normal function of the new valve-in-valve bioprosthesis was confirmed by transthoracic echocardiography (Figure 4I–L). The periprocedural course was uneventful. At 22 months follow-up, the patient is in NYHA functional class I with moderately reduced (yet significantly improved) RV function and normal function of the bioprosthesis.

### 3.3. Patient C

**Figure 5:**
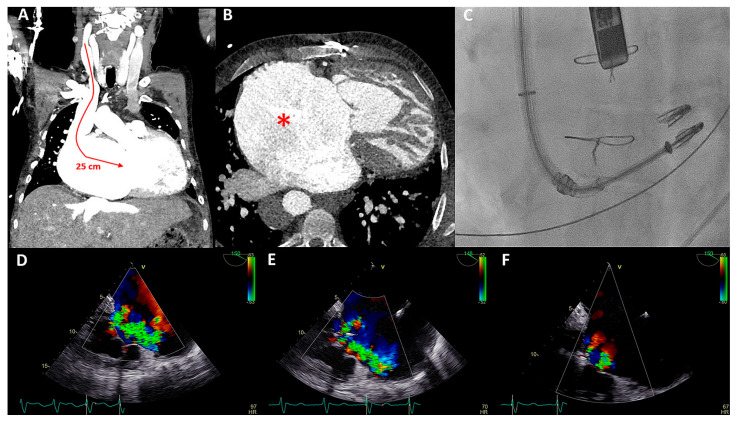
(**A**) Distance between the right internal jugular vein and tricuspid valve (TV) measured on a computed tomography (CT) scan in a coronal plane (arrow). (**B**) Severely dilated mono-atrium (asterisk) on CT scan in a sagittal plane. (**C**) Implantation of the second XTW clip (note the first XTW clip already released) under transesophageal echocardiography (TEE) guidance. (**D**) Preprocedural TEE imaging of the torrential (IV+) TR. (**E**) Periprocedural TEE showing TR after placing the first XTW clip. (**F**) TEE showing the significant reduction in TR after placing the second XTW clip to grade I–II.

A 48-year-old male born with complex cyanotic congenital heart disease (left isomerism, Fallot type double outlet right ventricle (DORV), mitral valve atresia, large ventricular septal defect (VSD), functionally univentricular heart with a hypoplastic left ventricle) was palliated using multiple Blalock–Taussig (BT) shunts. He had an abnormal systemic venous return with a hemiazygos continuation of the inferior caval vein (IVC) and a persistent left superior caval vein (SVC) (Figure 5A). The patient had multiple hospitalizations for heart failure with functional TR due to severely dilated functional mono-atrium and annulus (Figure 5B) and systemic right ventricular dilation with (moderately) impaired systolic function and a restrictive filling pattern. Laboratory analysis showed preserved renal function (eGFR 71 mL/min/1.73 m^2^) and elevated levels of NT-proBNP (5592 ng/L, upper reference limit 121 ng/L). TTE and TEE (Figure 5D) showed a severely dilated functional mono-atrium and RV with torrential TR. Computed tomography (CT) was performed for pre-procedural assessment of the approach (i.e., the trajectory and the distance from the right internal jugular vein to the TV was accessed as a potential venous access route given the anatomy). Furthermore, the CT showed a dilated ventricle of 12 × 11cm in the axial view and multiple large venous collaterals. Surgery was deemed high risk due to three previous thoracotomies, pronounced collaterals, and the severely dilated ventricle with reduced systolic function. Because of the hemiazygos continuation of the IVC, transcatheter access via the femoral vein was not feasible. Therefore, a transjugular TriClip (Abbott, IL, USA) transcatheter valve repair of the TV was performed under TEE guidance. Two XTW clips were implanted capturing the anterior and septal leaflets.

As a result of the severely dilated annulus and tethering, the coaptation gap was large, and simultaneous grasping of both leaflets was challenging. The TriClip system has the advanced option of independent leaflet grasping. First, the anterior leaflet (technically more challenging) was grasped and, subsequently, after minor device repositioning under TEE guidance, the septal leaflet was captured and the clip was released. The first clip was implanted near the commissure to narrow the coaptation gap so that the second clip could be implanted to treat the regurgitation (Figure 5C), which decreased to moderate (Figure 5E,F). Three weeks after the percutaneous procedure, at the outpatient clinic, the patient reported a decrease in orthopnea and exercise-induced dyspnea as the NYHA functional class was reduced from IV to III. At 17 months follow-up, the patient remained in NYHA functional class III. However, multiple heart-failure-related admissions occurred during follow-up (latest level of NT-proBNP was 8092 ng/L) and the patient developed atrial fibrillation (AF) which was treated with amiodarone.

### 3.4. Patient D

**Figure 6:**
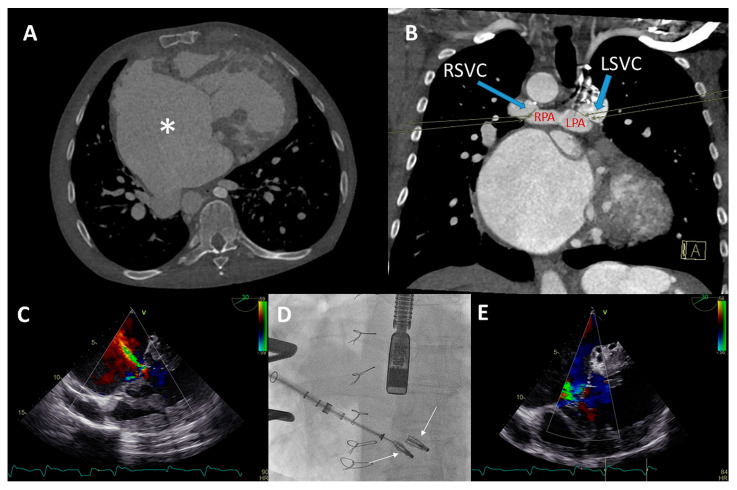
(**A**,**B**) Computed tomography (CT) scan of the thoracic cavity with the anatomical position of the dilated functional mono-atrium (asterisk) against the right thoracic wall (panel **A**) and bidirectional bilateral Glenn shunt (panel **B**) (LPA = left pulmonary artery, RPA = right pulmonary artery, LSVC = left superior vena cava, RSVC = right superior vena cava). (**C**) Pre-procedural transesophageal echocardiography (TEE) which visualized severe common atrioventricular (AV) valve regurgitation. (**D**) Anteroposterior fluoroscopic view of the two XTW MitraClips positioned in the AV valve. At the same time, it is appreciated that the delivery system is positioned through the fifth intercostal space after a right mini-lateral thoracotomy. (**E**) Moderate AV regurgitation after the hybrid procedure visualized with TEE.

A 52-year-old man was born with left isomerism and a functionally univentricular heart. There was a large ASD, common AV valve, DORV with transposition position of the great arteries (TGAs), pulmonary stenosis, hypoplastic left ventricle, a right-sided aortic arch, a persistent left SVC, and an interrupted IVC (azygos continuation). At the age of 30, he received a bilateral bidirectional Glenn shunt. The patient was now admitted with peripheral edema and permanent AF with adequate rate control and had preserved renal function (eGFR 83 mL/min/1.73 m^2^). Moderately reduced systolic and diastolic systemic ventricular function, severe atrial dilatation, and AV valve regurgitation were documented on TTE and TEE. Additional CT imaging showed a severely dilated functional mono-atrium in close proximity to the thoracic wall (Figure 6A), a patent bilateral SVC to pulmonary (Glenn) connection (Figure 6B), and an extensive network of coronary fistulae was noted. Subsequent catheterization confirmed connection of the azygos continuation of the IVC with the functional mono-atrium without a gradient, low pressures in the Glenn conduits, and moderate pulmonary stenosis. Cardiac decompensation with elevated levels of NT-proBNP (1314 ng/L, upper reference limit 121 ng/L) was adjudicated to the severe AV valve regurgitation and reduced systolic ventricular function (Figure 6C), and, despite escalating in diuretic treatment, the patient remained symptomatic (NYHA functional class III).

Conventional surgical AV valve replacement or repair was considered extremely high risk due to the anatomical relation between the atrium and the thoracic wall, the extensive coronary fistulae, and the reduced ventricular function. Percutaneous AV valve replacement or repair was deemed non-feasible by the transvenous route due to interruption of the IVC with azygos continuation and sharp angulation into the mono-atrium (for a transfemoral approach). Furthermore, a transjugular approach was not feasible because of the bilateral Glenn connection. Therefore, it was decided that a hybrid procedure under general anesthesia with direct atrial access using a MitraClip delivery system (Abbott, IL, USA) was the best strategy.

The congenital cardiothoracic surgeon performed a right (mini) lateral thoracotomy in the fifth intercostal space to expose the giant mono-atrium. A double-purse string suture was placed and an incision was made to create an opening using a Safari wire for guidance and stability. The interventional cardiologist then placed two XTW clips under TEE guidance (Figure 6D), resulting in reduction in AV valve regurgitation to grade II (Figure 6E). Post-procedural TEE showed grade II regurgitation with stable position of the clips. No peri-procedural complications occurred. The patient could be discharged with adequate heart failure medication with an improvement in NYHA functional class (II). Unfortunately, the patient died after 9 months due to progressive ventricular dysfunction, worsening of the AV valve regurgitation, and heart-failure-related multi-organ dysfunction (NYHA functional class IV at the latest admission).

### 3.5. Patient E

A 72-year-old female born with Tetralogy of Fallot (TOF) and having undergone three previous thoracotomies (Blalock–Thomas–Tausig shunt at the age of 4, correction of TOF at the age of 22, and a pulmonary homograft implantation at the age of 58 years), numerous ablation procedures for atrial arrhythmias after which permanent AF was accepted, and a pacemaker implantation due to a high-degree AV block was evaluated at the outpatient clinic due to progressive complaints of dyspnea on exertion and a recent admission with right-sided decompensation. She was in NYHA functional class IV despite escalation in diuretic treatment.

**Figure 7:**
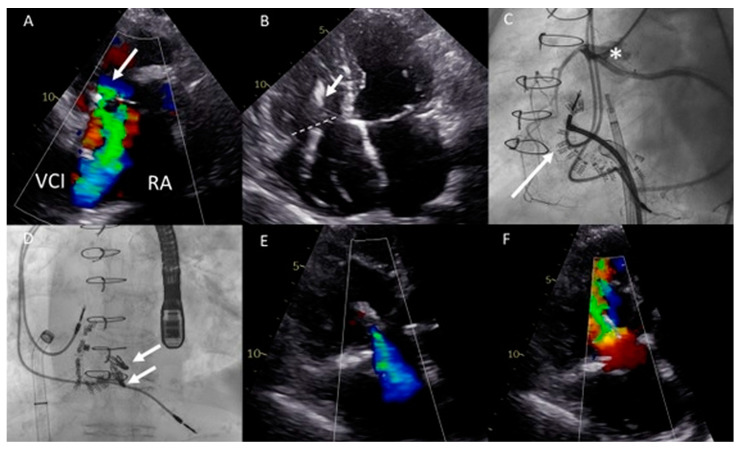
(**A**) Color Doppler modified parasternal right ventricular (RV) inflow view shows severe tricuspid regurgitation (TR) with a wide vena contracta (arrow) and leakage jet reaching the inferior vena cave (IVC). (**B**) Apical four-chamber view shows tricuspid valve (TV) annulus dilatation (dash line, 46 mm) and impingement by the RV pacemaker lead (arrow). (**C**) Left inferior oblique fluoroscopic view of the Cardioband (arrow) annulus reduction procedure (asterisk). (**D**) Anteroposterior fluoroscopic view shows the two XTW clips (arrows) implanted. (**E**,**F**) Modified apical four chamber color Doppler views showing mild (-moderate) residual TR and inflow through the TV (mean gradient of 3 mmHg).

Echocardiography showed severe TR with a regurgitant jet directed towards the inflow of the inferior vena cava and a progressive dilation of the RV with preserved systolic function (Figure 7A,B). The pulmonary homograft function was preserved. The mechanism of TR was considered to be multifactorial i.a. annulus dilatation in combination with impingement by the previous implanted RV pacing lead, leading to malcoaptation. The heart team deferred from surgical intervention on the TV due to high estimated peri-procedural risk in a septuagenarian with three previous thoracotomies. A two-step catheter-based approach was pursued: (1) Cardioband annulus reduction and (2) TriClip implantation to treat any hemodynamically significant residual TR.

Both procedures were performed in sequential order under general anesthesia, by means of right femoral access and fluoroscopy and transesophageal echocardiographic guidance (Figure 7C,D). A total of 15 anchors were used for the Cardioband annular reduction (Edwards Lifesciences, USA) reducing the annulus dimensions to 28 × 27 mm. Yet, moderate-severe TR persisted and the second procedure was planned for the patient. She underwent the placement of two TriClips (Abbott, IL, USA), resulting in reduction of TR to mild-moderate and a slightly elevated inflow gradient of 3 mmHg over de TV (Figure 7E,F). No periprocedural complications were documented. At 16 months follow-up, the patient remains in NYHA functional class II and is euvolemic. No heart-failure-related admissions occurred since the TV interventions.

## 4. Discussion

In this descriptive cohort, we report on the indication, methodology, and outcomes of transcatheter treatment of five consecutive ACHD patients with severe AV valve regurgitation in the setting of complex anatomical malformations: two patients underwent a TEER, one patient underwent a valve-in-valve procedure, one patient underwent a Cardioband system implantation, and one patient underwent a consecutive Cardioband implantation followed by TEER. In two out of five patients, the regurgitant AV valve was of the systemic ventricle (as opposed to three patients with AV valve regurgitation of the subpulmonary ventricle). Post-procedural AV valve regurgitation as well as NYHA functional class improved in all patients. This cohort is illustrative of the feasibility and the wide range of applications of transcatheter techniques in addressing AV valve lesions in ACHD patients and highlights the necessity of an individualized and anatomy-tailored approach.

### 4.1. Feasible and Patient Tailored Alternative to Surgery

All patients were discussed by a dedicated ACHD team and surgery was deemed to be too high risk for all patients reported. Lifetime rate of re-thoracotomies, extensive adhesions, unique anatomical challenges, hemorrhage prone collaterals, and multi-organ dysfunction are all recognized factors in this decision making favoring a transcatheter approach [9]. Many of these factors are not accurately accounted for by the EuroScore risk stratification and the validated ACHD specific cardiac surgery risk score is currently lacking. Shorter duration of hospitalization, avoidance of inflammatory response as a result of a cardiopulmonary bypass, and an overall shorter rehabilitation time are factors in favor of a transcatheter-based treatment.

However, despite the temptation of a less-invasive approach, it is important to recognize that data on procedural outcomes and long-term clinical results are scarce [5,6,7]. This calls for an international prospective registry addressing this gap in evidence in the heterogenous group of ACHD patients and a broad spectrum of valvular dysfunction. It is also important to be aware of the challenging vascular access sites and routes as well as specific intracardiac angulations (for which the standard delivery catheters are not optimized). The hemodynamic results of a (transcatheter) valve repair might not aways be as ideal as those of a replacement, and the tolerability and consequences of any residual lesions should always be considered in advance during heart team discussions. The increased risk of endocarditis after a majority of (right-sided) percutaneous interventions should also we weighed on a case-by-case basis.

The Cardioband system addresses the annular dilatation pathophysiology in a minimally invasive catheter delivered approach. Nickenig et al. conducted the TRI-REPAIR study in thirty patients with moderate or greater functional TR who received a Cardioband system. The implantation of a Cardioband resulted in sustained and significant TR reduction at two years follow-up with substantial improvement in NYHA functional class [10]. Gray et al. demonstrated similar results after one year follow-up [11]. To the best of our knowledge, the current manuscript describes for the first time the successful use of the Cardioband system to reduce TR in ACHD patients—one after numerous operations due to dysfunction of a bicuspid aortic valve and ascending aorta dilatation (patient A) and one late after TOF correction (patient E). The patients remain in NYHA functional class II 16 months and 2 years follow-up, respectively.

Valve-in-valve transcatheter TV replacement has previously been reported to be a good alternative to surgery in patients with severe TV regurgitation and high operative risk [12,13]. In line with this, we report a case of successful Edwards SAPIEN 3 valve implantation into a degenerated Sorin-Pericarbon MoreTM bioprosthesis in a patient with M. Ebstein, resulting in reduction of grade IV TR to trivial regurgitation and associated improvement in the NYHA class and RV function.

Furthermore, three patients underwent a successful TEER, where one patient had a systemic left ventricle whilst the two other patients had a systemic right ventricle. In particular, one of those patients had a functional mono-ventricle with reduced systolic function in the setting of a very complex anatomy, in which the implantation of the clips was feasible and safe (patient D). Despite best efforts, the patient died 9 months after the procedure due to progressive heart failure and concomitant multi-organ dysfunction. Ott et al. previously reported that TEER in congenitally corrected TGA was feasible and safe [14] and Schamroth Pravda et al. demonstrated successful TEER in ACHD patients with a range of primary defects [6].

Of particular interest for further studies remain the patients with a failing systemic right ventricle and TR (such as those late after Mustard/Senning repair or patients with congenitally corrected transposition of the great arteries), as well as the growing group of Fontan circulation patients with functional AV-valve regurgitation (REF + REF). These groups are at specifically high surgical risk and the potential of percutaneous AV valve therapies would be a welcome development to mitigate the risk of re-operations [15,16].

### 4.2. Dedicated ACHD Heart Team

The current series of patients illustrates the importance of a dedicated and experienced ACHD heart team in addressing AV valve dysfunction in this heterogenous patient group. Only after a thorough understanding of the mechanisms contributing to the valvular dysfunction, and careful consideration of the individual perioperative risks and the capability of a transcatheter technique in adequately addressing these mechanisms, can an individualized and weighted decision be made. Multidisciplinary expertise is essential and we advocate for all ACHD patients undergoing transcatheter AV valve repair or replacement to be discussed and treated in specialized centers by dedicated teams. As the vast majority of ACHD patients require a lifelong follow-up, the heart team can also be used as a forum to discuss treatment and follow-up strategies to ensure timely intervention (early enough to prevent irreversible remodeling and hemodynamic deterioration, yet late enough for the benefits of intervention to outweigh the lifetime risks of endocarditis and inevitable re-interventions).

### 4.3. Peri-Procedural Imaging and Potential for Virtual Reality

A comprehensive, multi-parametric, and multimodality approach is necessary to understand the underlying mechanism of AV valve regurgitation. This is essential to determine the optimal management strategy and timing of intervention. Given the current lack of robust data defining the optimal timing window for intervention for the vast majority of ACHD valvular lesions, and the often misleading seemingly “asymptomatic” status of the patients during long-term follow-up, serial imaging plays an essential role in timely detection of hemodynamic deterioration. Thorough review of patient records and focused imaging of the venous and arterial access and intracardiac connections is important, as many ACHD patients have concomitant vascular anatomy variants and venous return abnormalities and have undergone numerous vascular interventions, potentially compromising conventional vascular access routes. Patient C is illustrative of a transjugular venous access utilization and patient D required a hybrid procedure with surgical exposure of the functional mono-atrium and the common AV valve. Of note, several studies showed that pre-procedural planning using 3D modeling/virtual reality (VR) in pediatric cardiothoracic patients is feasible as there are some reports of the experience in ACHD patients [17,18,19]. VR utilization is increasing, with VR becoming more robust and accessible for daily practice [20]. VR is expected to play an important role in preprocedural planning and intraprocedural guidance, specifically for the ACHD cases with complex anatomy and challenging vascular access.

### 4.4. Study Limitations

This descriptive cohort is inherently limited by the small numbers of a heterogenous population and should therefore be interpreted in this context. It describes a two-center experience of an experienced ACHD interventional team and the techniques utilized might not be widely implemented in various settings. The follow-up period is limited and no data are available regarding the long-term (clinical) outcomes.

## 5. Conclusions and Clinical Implication

In conclusion, novel (hybrid) transcatheter valve therapies, including transcatheter edge-to-edge repair using a TriClip or MitraClip, transcatheter annulus reduction using a Cardioband, and valve-in-valve replacement techniques are feasible and can address AV valve regurgitation in highly selected complex ACHD patients. Comprehensive multimodality imaging and a dedicated ACHD heart team are essential in successfully implementing patient-tailored transcatheter techniques and deferring high risk surgery in selected patients. Long term data on hemodynamic and clinical outcomes are essential in evaluating the efficacy and durability of transcatheter AV valve interventions in ACHD.

## Figures and Tables

**Table 1:** Demographic and clinical patient characteristics at baseline and latest follow-up.

	Patient A	Patient B	Patient C	Patient D	Patient E
Age (years)	75	40	48	52	72
Sex	male	female	male	male	female
CHD diagnosis and surgical history	BAV with severe aortic regurgitation, Ross at the age of 25 and ascending aorta replacement and AVR bioprosthesis due to autograft failure at the age of 65	M. Ebstein with severe tricuspid regurgitation right-left shunt over an open PFO, TV annuloplasty followed by replacement with a bioprosthesis at the age of 31	Left isomerism, DORV-Fallot type, hypoplastic left ventricle, mitral valve atresia, right modified Blalock–Taussig shunt at the age of 1, left modified Blalock–Tausig shunt at the age of 13, aorto-pulmonary shunt to the right pulmonary artery at the age of 20 because of occlusion of the left Blalock–Taussig shunt	Left isomerism, DORV-TGA, large ASD, PS, right-sided aortic arch, persistent left SVC, bilateral bi-directional Glenn anastomosis at the age of 31	Tetralogy of Fallot, Blalock shunt at the age of 4 and surgical correction at the age of 22, pulmonary homograft implantation at the age of 58, numerous ablation procedures for atrial fibrillation and flutters, chronic RV pacing due to a high degree AV-block
Concomitant cardiac lesions and diagnoses	Paroxysmal atrial fibrillation	None	Multiple systemic to pulmonary artery shunts, abnormal venous return with hemiazygos continuation of the IVC and a persistent left SVC, paroxysmal atrial fibrillation	Persistence of left SVC, abnormal venous return with azygos continuation of the IVC, permanent accepted atrial fibrillation, endocarditis	Permanent accepted atrial fibrillation
Number of previous cardiac surgeries	2	2	3	1	3
Non-cardiac comorbidities	Bilateral pulmonary embolism, hypertenstion	-	-	Epilepsy	Epilepsy
Morphology of the systemic ventricle	left	left	right	right	left
Pre-procedural NYHA class	III	II	IV	III	IV
Morphology of the regurgitant AV valve	TV (subpulmonary)	TV (subpulmonary)	TV (systemic)	common AV-valve (systemic)	TV (subpulmonary)
Severity of AV valve regurgitation	IV+/torrential	IV/severe	IV+/torrential	IV/severe	IV/severe
(Dominant) mechanism of AV valve regurgitation	Annulus dilatation	Bioprosthesis degeneration	Annulus dilatation	Annulus dilatation	Annulus dilatation, impingement by device lead
Main imaging findings	Moderately reduced left and right ventricular function, dilated RV, severe/torrential TR and right atrial dilatation, estimated filling pressures 20 mmHg	Moderately reduced right ventricular function	Dilated ventricle with impaired systolic function, severely dilated functional mono-atrium	Severely dilated functional mono-atrium, extensive network of coronary fistulae, Moderalety reduced systolic and diastolic systemic ventricular function	Preserved right ventricular function
Renal function (eGFR)	63 mL/min/1.73 m^2^	85 mL/min/1.73 m^2^	38 mL/min/1.73 m^2^	>90 mL/min/1.73 m^2^	89 mL/min/1.73 m^2^
Cardiac pharmacotherapy	Vitamin K antagonist, sotalol, ACE-inhibitor, aldosterone receptor antagonist, loop diuretic, calcium antagonist, statin	None	Amiodarone, DOAC, aldosterone receptor antagonist, loop diuretic	Aldosterone receptor antagonist, DOAC, loop diuretic	Vitamin K antagonist, sotalol, aldosterone receptor antagonist, loop diuretic
Vascular access	Right femoral venous access	Right femoral venous access	Right transjugular venous access	Right lateral thoracotomy through the 5th intercostal space	Right femoral venous access
Intervention	Annuloplasty (Cardioband)	Valve-in-valve implantation (Sapien 3)	TEER (Triclip), two XTW clips between A-S leaflets	Hybrid TEER (MitraClip), two XTW clips between A-P leaflets	Annuloplasty (Cardioband) TEER (Triclip), two XTW clips
Procedural complications	None	None	None	None	None
Post-procedural AVVR grade	II	<I	II	II	I-II
Post-procedural NYHA class	I-II	I	III	II	II
NYHA class at latest follow-up	I-II	I	III	IV	II

ACE = angiotensin-converting enzyme; ASD = atrial septal defect; AVR = aortic valve replacement; AV = atrioventricular; AVVR = atrioventricular valve regurgitation; BAV = bicuspid aortic valve; CHD = congenital heart disease; DOAC = direct oral anticoagulants; DORV = double outlet right ventricle; eGFR = estimated glomerular filtration rate; IVC = inferior caval vein; NYHA = New York Heart Association; PFO = patent foramen ovale; PS = pulmonary stenosis; RV = SVC = superior caval vein; TEER = transcatheter edge-to-edge repair; TGA = transposition of the great arteries; TV = tricuspid valve.

## Data Availability

All data relevant to the study are included in the article. Additional data are available on reasonable request.
